# Religious and Spiritual Behaviors Correlated with Mental and Physical Well-Being Among American Muslims within the Twin Cities

**DOI:** 10.1007/s10943-026-02600-x

**Published:** 2026-03-16

**Authors:** Ashab Alamgir, Ahmad Zaro, Razi Lawabni, Fyzeen Ahmad, Juwayria Webo, Sashmitaa Alamgir, Marwa Saidi, Nea Nwanokwale, Mirza Baig, Muhab Abdelwahab, Ali Hassan, Amy Taylor

**Affiliations:** 1 University of Minnesota Medical School, Twin Cities, Minneapolis, MN USA; 2 University of Minnesota, Twin Cities, Minneapolis, MN USA; 3 University of Minnesota School of Nursing, Minneapolis, MN USA; 4 Bakken Center for Spirituality & Healing, University of Minnesota, Twin Cities, Minneapolis, MN USA

**Keywords:** Religion, Islam, Spirituality, Wellbeing, Religious practice

## Abstract

Previous studies have demonstrated the significant association between religiosity and spirituality (R/S) with well-being. However, little is known if a similar association exists between the religiosity and mental well-being within Muslims in U.S.-based R/S health research, who remain comparatively underrepresented relative to Judeo-Christian samples. This study assesses whether these associations hold true in this population and if they can be explained by demographic factors alone. A total of 337 participants were enrolled from Muslim community centers in the Minneapolis–St.Paul, Minnesota, metropolitan area (completion rate = 47.5%). Participants completed the Muslim Religiosity Scale (MRS) with intrinsic and behavioral subscales. They also completed physical and mental health questionnaires: Health-promoting Lifestyle Profile Questionnaire (HPLP II), General Anxiety Disorder 2 (GAD-2), and Patient Health Questionnaire 2 (PHQ-2). Associations were assessed using Spearman correlations and ordinary least squares regression. A statistically significant positive association was observed between MRS and HPLP Total Score (*ρ* = 0.35, *p* < .001). Lower MRS scores were associated with higher depressive symptoms (PHQ-2; *ρ* = − 0.28, *p* < .001) and higher anxiety symptoms (GAD-2; *ρ* = − 0.29, *p* < .001). Associations remained significant after adjustment for age, sex, race/ethnicity, and income (*p* < .01). Associations were stronger for the behavioral MRS subscale (e.g., prayer and service attendance) than the intrinsic subscale. Generalizability may be limited by English-only administration and incomplete responses. This may overrepresent more English-proficient, more educated, and more health-engaged participants and bias estimates. This study may inform future efforts to improve cultural sensitivity, pastoral care, and patient-provider relationships in spiritually diverse populations.

## Introduction

Healthcare models have long understood the importance of lifestyle modifications, such as proper diet and exercise, for overall well-being. Patients with chronic diseases are often well-informed of the transformative benefits of moving a bit more or eating healthier. Despite this, healthcare providers often interact with patients who unfortunately seem to have lost motivation in the face of multidimensional medical, social, financial, and personal challenges. Recent studies have shown that the prevalence of negative mental health symptoms among patients with terminal diagnoses can be up to 50%, particularly among those with short-term prognoses (Huey et al., 2018; Lee et al., 2021; Mitchell et al., 2011). These negative mental health symptoms have been shown to be a complicating factor in the care continuum from diagnoses to treatment due to a multitude of factors such as poor treatment adherence, hormonal dysregulation, and more that has been shown to increase hospitalization, worsen morbidity, and reduce survival rates (Andersen et al., 2023; Mossman et al., 2021; Sobel & Markov, 2005). It remains apparent that a critical barrier to providing patient care is understanding what motivates patients to be healthy, even in the face of seemingly insurmountable circumstances. A comprehensive care model with improved understanding and proactive management of these factors may enhance care delivery and patient outcomes.

Among several factors, religiosity and spirituality (R/S) have been previously cited as key factors in improving resilience and the overall well-being of patients faced with medical and mental health challenges (Hill & Pargament, 2003; Nagy et al., 2024; Ventis, 1995). This association holds true not only for members of Abrahamic faiths such as Muslims, Christians, or Jewish individuals, but also for practitioners of ancestral religions such as Buddhism or Shintoism (Nagy et al., 2024). For instance, a 2024 meta-analysis found that incorporating religion and spirituality into cancer care significantly improved patients’ ability to cope and reduced depressive symptoms (Jim et al., 2015; Nagy et al., 2024). Similarly, individuals who displayed higher levels of religious coping behaviors had a lower risk of anxious and/or depressive symptoms during the COVID-19 pandemic (Thomas & Barbato, 2020). Furthermore, previous studies found that psychotherapy that incorporated religion and spirituality was more effective in preventing depressive and anxious symptoms for religious individuals, especially young patients (Aggarwal et al., 2023; Ventis, 1995). Finally, religious individuals are often well integrated into social networks that provide an additional layer of support against well-established risk factors for mortality such as loneliness (Murthy, 2023).

There exist several hypothesized mechanisms behind these well-established associations. From a physical health perspective, many religions encourage healthy habits such as fasting, abstention from alcohol, smoking, or other recreational drugs (Livne et al., 2021; Pawlikowski et al., 2019). Furthermore, many discourage eating more than necessary and often prescribe simple ways of life devoid of excess (Borges et al., 2021; Chen et al., 2021). Additionally, religions often promote several beliefs and practices that may aid in mental well-being. For instance, religions often universally promote acts of service and practicing active gratitude. Moreover, concepts like purpose/destiny, divine justice, and reward for persistence in the face of suffering may serve as powerful protective factors that enable individuals to more easily relinquish emotional insults, hold onto hope, and insulate themselves against despair even against overwhelming physical or social circumstances (Aksoy et al., 2022; Krause et al., 2010). Despite a growing recognition of this dimension of clinical care, existing research on the association between religiosity and health remains limited in scope, particularly with respect to underrepresented faith groups like Islam (Ali et al., 2025; Hall et al., 2008). Understanding how religion and/or spirituality may play an integral role in patients’ well-being will allow practitioners to better understand their patients, improve cultural sensitivity, and ultimately strengthen the physician–patient relationship (Piracha et al., 2024; Sarsour & Hammoud, 2021).

Islam, the fastest growing religion globally, with projections to become the largest faith group by the end of the century, represents a significant and increasingly influential religious community (Lipka & Hackett, 2017). Yet, the majority of studies on R/S in the context of health focus on Judeo-Christian populations, while Muslims, particularly those in the USA, remain largely underrepresented in these studies (A. Padela & Raza, 2015). Thus, previously observed results may largely be driven by non-Muslim, majority religious groups. This may contribute to a poor understanding of Muslim patients and existing healthcare disparities (A. I. Padela & Zaidi, 2018; Piracha et al., 2024). Thus, this study assesses the nature of the relationship between R/S and health among a major Muslim population within the USA.

### Research Questions:

1. What evidence is there of an association between markers of physical and/or mental health with markers of religious and/or spiritual practice?
2. Do demographic confounders like income, status, or race account for the observed associations?
3. Are these associations more strongly associated with religious identity, or behavioral factors such as prayer or religious service attendance?

## Materials and Methods

### Study Design and Participants

Adult (> 18 years) Muslims were surveyed at seven sites around the Twin Cities, including mosques, the University of Minnesota, and a Saturday school between February and May 2025. Study staff set up an information table after Friday sermons or after Saturday school. Adults were invited to participate and were provided with QR codes linking to a REDCap survey completed on personal devices. Only respondents who were consented and completed the entire survey were included in the analysis. In total, 337 participants completed the survey. The survey was administered in English only, and participation was voluntary and anonymous.

### Measurements and Data Collection

The survey collected data on demographics and validated measures of religiosity, health-promoting behaviors, resilience, depression, and anxiety.

### Demographics

In the demographic section, participants reported biological sex, age, household income, and race/ethnicity.

### Muslim Religiosity Scale (MRS)

Religiosity was assessed with the MRS (Al Zaben et al., 2015; Mahudin et al., 2016). We followed recommended best practices for the use of validated R/S scales and provide full English item wording with response options in Appendix 1 (Koenig & Al Zaben, 2021). Behavioral items in this scale assess practices such as frequency of daily prayer, mosque attendance, and Qur’an reading, while intrinsic belief items assess perceptions such as the presence of Allah in daily life and the extent to which faith guides personal decisions. Most questions use a response scale ranging from 1 to 5, though the specific scale varied by item. Scores for each item were summed to produce two subscale scores: Religious Practices (range: 10–50, higher scores indicating greater behavioral engagement) and Intrinsic Religiosity (range: 3–15, higher scores indicate stronger intrinsic belief). The total MRS score, ranging from 13 to 65, reflects the combined sum of these two subscales, with higher scores representing greater overall religiosity.

This scale was selected with deliberate attention to the growing recognition of contamination in R/S measures. Contamination of R/S measures is the inadvertent inclusion of items that assess mental or social health indicators (e.g., meaning, purpose, peace, or well-being) rather than strictly religious constructs (Koenig & Carey, 2024). Such contamination can confound associations between religiosity and health outcomes by introducing tautological relationships. To mitigate this risk, the present study utilized the MRS, a psychometrically validated instrument specifically designed to capture religious belief and behavior independent of emotional or social functioning. The MRS operationalizes religiosity through distinct behavioral (e.g., prayer, Qur’an reading) and intrinsic (e.g., faith-guided decision-making, perception of Allah’s presence) domains that are conceptually uncontaminated by psychological or social well-being constructs (Al Zaben et al., 2015; Mahudin et al., 2016). In addition, many analyses in this study examined the two subscales separately rather than relying solely on total scores. This analytic approach aligns with best practices for R/S measurement, which emphasize the examination of subscales individually to prevent interpretive distortions that can arise when contaminated components are aggregated (Koenig & Carey, 2025).

### Health-Promoting Lifestyle Profile II (HPLP II)

Health-promoting behaviors were measured with the HPLP II, a validated instrument that included 52 items (Walker et al., 1995). Respondents indicated how frequently they engaged in each behavior using four categories: never, sometimes, often, and routinely (numerically scored 1 to 4 in this order). Traditionally, HPLP II scores are reported as means on a 1–4 scale. In this study, item responses were summed. As a result, the Total score ranged from 52 to 208. Subscale ranges were as follows: Health Responsibility, 9–36; Physical Activity, 8–32; Nutrition, 9–36; Spiritual Growth, 9–36; Interpersonal Relations, 9–36; and Stress Management, 8–32. Higher scores reflect more frequent health-promoting behaviors.

### Brief Resilience Scale (BRS)

Resilience was assessed with the BRS, a validated tool that comprises six questions rated on a Likert-style scale (Smith et al., 2008). Scores are averaged to yield a value from 1 to 5, with higher values indicating greater resilience.

### Patient Health Questionnaire-2 (PHQ-2)

Depressive symptoms were screened using the PHQ-2. This validated measure asked participants to indicate how often in the past two weeks they had little interest or pleasure in doing things and how often they felt down, depressed, or hopeless. Response options were not at all (0), several days (1), more than half the days (2), and nearly every day (3). The two items were summed to produce a total score from 0 to 6, with higher scores indicating greater depressive symptom burden.

### Generalized Anxiety Disorder-2 (GAD-2)

Anxiety symptoms were screened using the validated and reliable GAD-2. Participants reported how often they felt nervous, anxious, or on edge and how often they were unable to stop or control worrying, using the same four response options as the PHQ-2, scored 0–3. Scores were summed to produce a total from 0 to 6, with higher scores indicating greater anxiety.

## Statistical Analysis

Of 709 individuals who initiated the REDCap survey, 337 completed all items and were included in analyses (completion rate = 47.5%); 372 (52.5%) did not complete the survey and were excluded. Analyses were conducted using complete surveys only. To assess the relationship between measures of religiosity and well-being, we utilized two analytical approaches. First, we computed Spearman’s rank-order correlations to evaluate the strength and direction of bivariate associations. Second, we conducted ordinary least squares (OLS) regression analyses to estimate the magnitude and direction of these associations, reporting OLS regression coefficients (*β*) and R-squared values. All analyses were initially conducted using unadjusted models. These models were then extended to adjust for key sociodemographic covariates, including age, sex, race/ethnicity, and income.

Bivariate associations among MRS (Total, Intrinsic, Practices), HPLP II (Total and six subscales), BRS, PHQ-2, and GAD-2 were assessed using Spearman correlation statistics as described above. Multiple linear regression models were fit to adjust for age, sex, income, and race/ethnicity. Statistical significance threshold was *p* < 0.05.

Preprocessing and summary statistics were computed in R (version 4.4.3). Main analyses were completed using Python and associated packages, including pandas, NumPy, Matplotlib, statsmodels, SciPy, and scikit-learn. All code is available in the following GitHub repository: https://github.com/fyzeen/divine_wellness.

### Ethical Procedures

Ethics approval was obtained from the Institutional Review Board of a university prior to data collection. Verbal informed consent was obtained by a study team member before participants accessed the survey. Participation was voluntary and anonymous.

## Results

Participant characteristics are summarized in Table 1.

**Table 1:** Distribution of age, sex, income, and race/ethnicity among participants (*N* = 337)

Variables	*n*	% of Sample
Sex		
Male	248	73.6
Female	89	26.4
Age		
18–24	120	35.6
25–34	67	19.9
35–44	61	18.1
45–54	50	14.8
55–64	30	8.9
65 +	9	2.7
Household Income		
0–40 k	48	14.2
40–75 k	44	13.1
75–100 k	52	15.4
100–150 k	79	23.4
150–250 k	56	16.6
250–500 k	34	10.1
500 k +	9	2.7
Did Not Respond	15	4.5
Race/Ethnicity		
White	60	17.8
Black or African American	133	39.5
American Indian or Alaska Native	2	0.6
Asian	120	35.6
Did Not Respond	22	6.5

Descriptive statistics for all scale scores are summarized in Table 2.

**Table 2:** Mean ± SD scores for the MRS, MRS subscales, HPLP II, HPLP II subscales, BRS, PHQ-2, and GAD-2 scales

Variables	Mean ± SD
MRS Total	37.13 ± 3.91
MRS Intrinsic Religiosity Subscale	3.98 ± 1.51
MRS Religious Practices Subscale	33.15 ± 4.28
HPLP II Total (Health-promoting Lifestyles)	140.77 ± 21.28
HPLP II Health Responsibility Subscale	21.52 ± 5.34
HPLP II Physical Activity Subscale	19.45 ± 5.23
HPLP II Nutrition Subscale	23.12 ± 4.30
HPLP II Spiritual Growth Subscale	28.81 ± 4.34
HPLP II Interpersonal Relations Subscale	26.94 ± 4.72
HPLP II Stress Management Subscale	20.93 ± 4.09
BRS	3.09 ± 0.40
PHQ-2	1.17 ± 1.44
GAD-2	1.26 ± 1.59

### Religiosity is Significantly Associated with Health-Promoting Behaviors

Higher religiosity, as measured by the Muslim Religiosity Scale, was significantly and positively associated with multiple domains of health behavior, as measured by the Health-Promoting Lifestyle Profile (HPLP). There was a statistically significant association between the HPLP Total Score and MRS (*ρ* = 0.348, *p* <.001; Table 3).

**Table 3:** Partial Spearman correlations (*ρ*) between Muslim religiosity and well-being outcomes, adjusted for age, sex, race/ethnicity, and income (*N* = 337) *: *p* < 0.05, **: *p* <.01, ***: *p* <.001

Measure	MRS practices	MRS intrinsic	HPLP II total	PHQ-2	GAD-2	BRS
MRS total	0.968***	0.608***	0.301***	− 0.179***	− 0.172**	0.032
MRS practices subscale	–	0.430***	0.300***	− 0.161**	− 0.196***	− 0.005
MRS intrinsic subscale	–					
	–	0.206***	− 0.185***	− 0.058	0.086	

The strongest associations between MRS and HPLP subscores were observed for spiritual growth (*ρ* = 0.367, *p* <.001), nutrition (*ρ* = 0.303, *p* <.001), and interpersonal relations (*ρ* = 0.272, *p* <.001). These associations remained statistically significant in covariate-adjusted OLS models (Table 4).

**Table 4:** Adjusted OLS regression results for Muslim Religiosity Scale (MRS) Total Score predicting well-being outcomes (*N* = 337)

Outcome	MRS total *β*	MRS total *p*	MRS total R^2^
HPLP II Total (Health-promoting Lifestyles)	1.29	< 0.001	0.12
HPLP II Health Responsibility	0.25	< 0.001	0.07
HPLP II Physical Activity	0.11	0.08	0.02
HPLP II Nutrition	0.23	< 0.001	0.09
HPLP II Spiritual Growth	0.28	< 0.001	0.14
HPLP II Interpersonal Relations	0.22	< 0.001	0.08
HPLP II Stress Management	0.2	< 0.001	0.08
Brief Resilience Scale (BRS)	0.03	0.15	0.01
PHQ-2	− 0.07	< 0.001	0.07
GAD-2	− 0.08	< 0.001	0.08

Models adjusted for age, sex, race/ethnicity, and income. *β* = OLS regression coefficient. R^2^ = model R-squared

Muslim religiosity was also inversely associated with indicators of mental health burden. Higher MRS was associated with fewer symptoms of depression (PHQ: *ρ* =  − 0.281, *p* <.001; Table 3) and anxiety (GAD: *ρ* =  − 0.293, *p* <.001; Table 3). In covariate-adjusted OLS models, higher MRS was associated with lower PHQ-2 and GAD-2 scores (Table 4).

No significant relationship was observed between MRS and psychological resilience, as measured by the BRS (*ρ* = 0.047, *p* >.05; Table 3). This suggests that the protective mental health effects of religiosity may not also improve psychological resilience.

To assess the magnitude of these associations and the variance in well-being explained by religiosity, we conducted adjusted OLS regression analyses. Higher MRS total score was associated with higher HPLP II total score (*β* = 1.29, *p* <.001, R^2^ =.12) and lower PHQ-2 (*β* = − 0.07, *p* <.001, R^2^ = 0.07) and GAD-2 scores (*β* = − 0.08, *p* <.001, R^2^ = 0.08); no association was observed with BRS (*p* = 0.15) (Table 4).

### Muslim Religious Practices are More Strongly Associated with Health than Intrinsic Religiosity

Analysis of the MRS subscales revealed an interesting pattern: religious practices are more strongly and consistently associated with health than intrinsic religiosity, even after controlling for demographic covariates (Table 5).

**Table 5:** Adjusted OLS regression results for MRS subscales predicting well-being outcomes (*N* = 337)

Outcome	MRS practices *β*	MRS practices *p*	MRS practices R^2^	MRS intrinsic *β*	MRS intrinsic *p*	MRS intrinsic R^2^
HPLP II Total (Health-promoting Lifestyles)	1.52	< 0.001	0.12	2.82	< 0.001	0.05
HPLP II Health Responsibility	0.3	< 0.001	0.08	0.45	0.004	0.02
HPLP II Physical Activity	0.16	0.028	0.02	− 0.07	0.87	0
HPLP II Nutrition	0.28	< 0.001	0.1	0.38	0.005	0.02
HPLP II Spiritual Growth	0.29	< 0.001	0.11	0.94	< 0.001	0.12
HPLP II Interpersonal Relations	0.24	< 0.001	0.06	0.73	< 0.001	0.06
HPLP II Stress Management	0.24	< 0.001	0.08	0.38	0.005	0.02
Brief Resilience Scale (BRS)	0.03	0.24	0	0.11	0.15	0.01
PHQ-2	− 0.07	< 0.001	0.06	− 0.2	< 0.001	0.05
GAD-2	− 0.1	< 0.001	0.1	− 0.09	0.08	0.01

For example, in the bivariate correlations (Table 3), intrinsic religiosity was associated with HPLP II total score (*ρ* = 0.206), but this was weaker than the association for religious practices (*ρ* = 0.300). These trends persisted among the HPLP subscales. For example, religious practices were significantly associated with physical activity (*ρ* = 0.152, *p* <.01), nutrition (*ρ* = 0.236, *p* <.001), and stress management (*ρ* = 0.264, *p* <.001). By contrast, intrinsic religiosity was not significantly correlated with physical activity (*ρ* = 0.029, *p* >.05), and its association with other HPLP subdomains was weaker (e.g., Nutrition: *ρ* = 0.128; Stress Management: *ρ* = 0.125).

Additionally, both subscales were negatively associated with depression and anxiety symptoms, with larger associations for religious practices (PHQ-2: *ρ* = − 0.161; GAD-2: *ρ* = − 0.196) than intrinsic religiosity (PHQ-2: *ρ* = − 0.185; GAD-2: *ρ* = − 0.058) (Table 3).

These findings suggest that the behavioral aspects of Muslim religiosity (e.g., attending prayer, fasting, or engaging in other religious practices) may exert a more tangible influence on health-promoting behaviors and emotional regulation than purely internal or belief-oriented dimensions of Islamic faith.

## Discussion

### Principal Findings

This study examined the relationship between R/S and overall well-being among a population of Muslims in a large metropolitan U.S. city, contributing the perspective of a previously underrepresented but rapidly growing population to a growing global literature landscape on R/S (Lipka & Hackett, 2017; A. Padela & Raza, 2015). Our results indicate concordance with previous studies that show that R/S correlates with markers of both physical and mental well-being, as displayed in the positive correlation with physical health markers like the MRS with HPLP II, and inverse correlation with negative markers of mental health like the PHQ and GAD (Aksoy et al., 2022; Nagy et al., 2024; Thomas & Barbato, 2020). Furthermore, these trends hold true even after adjusting for important demographic variables like age, sex, or income.

### Comparison with Prior Literature

As demonstrated by the MRS subscale analysis, our study also demonstrates that much of the positive effects come from religious participation and behavior (e.g. meditation or attending religious gatherings), rather than simply holding a religious identity. This finding aligns with those of a previous review that found greatly reduced risk of suicide, depression, and all-cause mortality among individuals who regularly attended religious services (Li et al., 2016). Studies have noted that the observed benefit of R/S was weaker for those who simply identified with a particular faith, but did not regularly partake in religious behaviors or gatherings (Musick et al., 2004; VanderWeele, 2017).

There are several mechanisms that may explain the positive effects of R/S on overall well-being. Firstly, sociological studies have observed religious communities to be significantly longer lasting and closer knit when compared to communities based on secular commonalities alone such as hobbies or profession (Sosis & Bressler, 2003). For instance, Muslims believe themselves to be a part of a larger community (*Ummah*), which provides a universal social fabric and sense of global community (Denny, 1977). Furthermore, deeply emphasized community events like weekly Friday prayer (Jummah) provide regular support for this sense of social identity (Dana et al., 2011; Nguyen, 2017; Saraei et al., 2024). In light of the 2024 U.S. Surgeon general’s report on the epidemic of loneliness and the drastic health benefits of community, it is plausible that the observed benefits in this study are largely explained by community (Debnam et al., 2011; Murthy, 2023). However, other studies have noted that the strength of the health benefits seen in religious communities is not explained by community alone, indicating that factors beyond community such as R/S may be independently responsible (VanderWeele, 2017).

### Potential Mechanisms

To illustrate, many religions promote the belief in a larger purpose and meaning in hardship that may allow individuals to think beyond their perceived circumstances (Krok, 2015). This may help explain the insulation against despair, depression, and suicidal tendencies found in this study and previous ones (Aggarwal et al., 2023; Brandão, 2025; Thomas & Barbato, 2020; Ventis, 1995). This has been further demonstrated in a review which found that religious individuals have higher rates of emotional regulation conducive to well-being (Brandão, 2025). Muslims hold a strong belief in a divine plan (Qadr), and are actively taught that if they remain patient and persevere through hardship they will find ease and be rewarded for it, whether in this life or the next—built on a trust that God will guide one through any circumstance (Tawakkul) as demonstrated in Exhibit 1. Similarly, belief in a day of judgement may allow religious individuals to relieve themselves of negative emotions such as resentment or anger during perceived injustices in the belief that they will inevitably be compensated for it.

In another vein, Muslims organize their day around five obligatory daily prayers, beginning with an early morning prayer and also ablution/washing (Wudu) prior to each prayer. This provides a predictable daily cadence to one’s day that protects against a loss of routine, maintains basic hygiene, and regularly reaffirms a connection with the divine—all three of which may protect against negative mental health symptoms (de Sá Couto-Pereira et al., 2024). Finally, Muslims must give a minimum 2.5% of their wealth to charity every year (Zakat), and are encouraged to give nonobligational charity (Sadaqa) year-round. Altogether, these behaviors reinforce habits of altruism, social integration, and gratitude which have been strongly linked to better social and mental well-being (Diniz et al., 2023). Ultimately, these religious and spiritual behaviors provide a strong sense of personal identity that may help ground individuals psychologically.

“Wondrous is the affair of the believer for there is good for him in every matter and this is not the case with anyone except the believer. If he is happy, then he thanks Allah and thus there is good for him, and if he is harmed, then he shows patience and thus there is good for him.”

Exhibit 1: Sahih Muslim 2999.

#### Health Behavior Pathways

Aside from the mental and social benefits of R/S, in many cases religions provide direct guidance on physically healthy behaviors. This often includes dietary guidance, fasting, basic hygiene, abstaining from harmful substances, and more. Relevant to this study, Muslims regard their bodies as a gift entrusted to them (Amanah) from God, and thus Muslims believe in an obligation to take care of them. This hinders the urge to do with one’s own body as one pleases, even if that behavior is unhealthy. This religious construct of the body being an entrusted gift may impede pleasurable but unhealthy behaviors that may be more often seen in secular paradigms and societies. For instance, it is well known that tobacco and alcohol are cited by the World Health Organization (WHO) as the first and third leading causes of global disease burden and many religions, particularly Islam, prohibit both (Singer et al., 2011). This has been observed in lower rates of both smoking, drinking, or recreational drug use among religious individuals and their associated health benefits (Chagas et al., 2023; Gillum, 2005). Furthermore, Muslims practice chastity and limit sexual activity to marriage, reducing rates of sexually transmitted infections when compared to secular counterparts (Gillum & Holt, 2010; Obeid et al., 2024). Finally, there is a large emphasis on avoiding dietary excess within many religions, along with prescribed fasting for extended periods (e.g. Ramadan), which has been correlated positively with several markers of metabolic and cardiovascular health (Gholampoor et al., 2024).

#### Clinical Implications

In the context of healthcare, this study aims to ultimately improve respect for the role of patients’ religious and spiritual identities in helping them cope with difficult physical and mental health diagnoses. Previous large-scale reviews have demonstrated that, when welcomed by patients, therapy that incorporates traditional elements of religiosity and spirituality improves outcomes for patients with difficult diagnoses (Balboni et al., 2022). Our study aligns with these findings by demonstrating that the behavioral element of health has a strong association with mental and physical health markers. This highlights the importance of pastoral care, and for healthcare providers to take a spiritual history using established tools such as the Faith, Importance and Influence, Community, and Address (FICA) Spiritual History questionnaire, and collaborate with chaplaincy, when applicable (Borneman et al., 2010). This will allow providers to not only better understand their patients’ motivational state, but also to advance their care as these resources may have an impact on health independent of other demographic or treatment-related variables. For instance, providers in the Twin-Cities may work with the large local Muslim-Somali patient population to explore habit stacking by combining medication adherence, sleep, or exercise around other deeply held and revered habits like five daily prayers.

## Limitations

While this study has notable findings, it also has several limitations. Firstly, this study relies on self-report which may result in a social desirability bias, even though the responses were collected anonymously (Podsakoff et al., 2003). In addition, the survey completion rate was 47.5% (337 of 709 individuals who initiated the survey), which raises the possibility of nonresponse bias if those who completed the survey differed systematically from those who did not. For example, individuals who are more engaged in health-promoting behaviors, more educated, and/or more proficient in English may have been more likely to complete the survey, which could bias the observed associations and limit generalizability (Fry et al., 2017; Wenz et al., 2021). Furthermore, given the cross-sectional design of this study, it is not possible to draw definitive conclusions about causality between R/S and health behaviors. Also, this study was predominantly composed of mosque-going individuals that may prioritize behavioral aspects of religion, which limits generalizability to non-mosque-going individuals that may be less religious. Finally, this study was limited to English speakers which may have precluded certain individuals, often older immigrants, from partaking in the study. As a result, while this study fits with the overall trend, further research that is longitudinal in nature with a larger sample size is needed. Furthermore, future studies should be done in agnostic/atheist populations to provide a comparative baseline.

## Conclusion

The findings in this study show that markers of spirituality and religiosity are significantly associated with positive physical and mental health status among Muslims in the Twin Cities area. Interestingly, these observed positive effects were more strongly correlated with religious and spiritual behaviors such as prayer, attendance at religious events, and meditation rather than religious belief or identity without concurring behavior. Thus, it may help inform a larger effort within the U.S. healthcare system to strengthen the provider–patient relationship and understand the factors that inspire patient well-being, particularly for those faced with complex biopsychosocial challenges through pastoral care or spiritual history taking. This study design should be replicated in other faith-based communities to improve understanding of patients from diverse religious and spiritual backgrounds.

## Appendix 1 Survey Instruments

### Demographic Data

See Table 6.

**Table 6:** Demographic questionnaire items and response options

Item #	Question	Response Options
1	What is your biological sex?	Male; Female
2	How old are you?	Drop Down Menu
3	What is your household income level?	0–40 k; 40–75 k; 75–100 k; 100–150 k; 150–250 k; 250–500 k; 500 k +; Prefer not to answer
4	What is your race/ethnicity?	White; Black or African American; American Indian or Alaska Native; Asian; Native Hawaiian or Other Pacific Islander; Prefer not to answer

### Muslim Religiosity Scale (MRS)

See Table 7.

**Table 7:** MRS items and response options

Item #	Question	Response Options
1a	How long have you been a Muslim? (Since birth)	No; Yes
1b	If no, how many years have you been a Muslim?	Open numerical response (0 if not Muslim)
2	How often do you attend group religious services for worship and prayer at Mosque or in small group (Fard)?	5 times/day; 1–4 times/day; Several times/week; Several times/month; Never
3	How often do you pray alone in private (Nawafl)?	Never; Rarely; Occasionally; Often; Very often
4	Are you regular in prayer or do you sometimes sum 2 or more obligatory prayers or skip?	Always skip; Often skip; Sometimes skip; Occasionally skip; Never skip (Regular)
5	How often do you read or recite the Qur’an or other religious literature?	Not at all/rarely; Ramadan only; Occasionally (< several times/week); Several times/week; Once/day or more
6	How often do you listen to or watch religious programs on radio or TV?	Not at all/rarely; Ramadan only; Occasionally (< several times/week); Several times/week; Once/day or more
7	Do you give Zakat to poor each year?	Never; Rarely; Occasionally; Often; Very often
8	Do you give money to poor as a free gift (not obligatory like Zakat)?	Never; Rarely; Occasionally; Often; Very often
9	How often do you fast (Sawm)?	Never; Ramadan (part month); Ramadan (all month); Ramadan (all month) + Occasional Nawafl; Ramadan (all month) + Many Nawafl
10	How often do you make Hajj?	Never; Once; Twice; Several times (not yearly); Yearly
11	How often do you make Umrah?	Never; Once; Several times; Every year; Several times per year
12	In my life, I experience the presence of Allah/God	Definitely true; Tends to be true; Unsure; Tends not true; Definitely not true
13	My religious beliefs lie behind my whole approach to life	Definitely true; Tends to be true; Unsure; Tends not true; Definitely not true
14	I try to carry my religion over into all other dealings in life	Definitely true; Tends to be true; Unsure; Tends not true; Definitely not true
